# Systemic Lupus Erythematosus With Multi-Organ Involvement in a Young Female: Lymphadenopathy, Lupus Cerebritis, Lupus Nephritis, and Cardiac Manifestations

**DOI:** 10.7759/cureus.15517

**Published:** 2021-06-08

**Authors:** Owaise Muhammad, Himanshu Jindal, Medha Sharath, Aadil M Khan, Sarang Choi

**Affiliations:** 1 General Medicine, Lugansk State Medical University, Kyiv, UKR; 2 Internal Medicine, Ganesh Shankar Vidyarthi Memorial Medical College, Kanpur, IND; 3 Internal Medicine, Bangalore Medical College and Research Institute, Bangalore, IND; 4 Internal Medicine, The Medical City, Pasig City, PHL

**Keywords:** systemic lupus erythematosus, anti-ds dna, ana, lupus nephritis, lupus cerebritis, lymphadenopathy

## Abstract

Systemic lupus erythematosus (SLE) is a multisystemic autoimmune disease that can affect almost every organ in the body. Its complications can often be fatal. The fatal complications include lupus cerebritis, lupus nephritis, and cardiac manifestations such as pericardial effusion. In this report, we discuss the case of a 23-year-old female who presented with complaints of high-grade fever, seizures, and altered mental state (AMS) and was found to have generalized lymphadenopathy (LAP). Various blood and urine analyses and radiological findings (chest X-ray, MRI of the head) were suggestive of lupus nephritis, lupus cerebritis, massive pericardial effusion, and thrombocytopenia. Her anti-double stranded DNA (anti-dsDNA) antibody was positive, and her pericardial fluid was positive for anti-nuclear antibodies (ANAs). She was administered IV glucocorticoids and phenytoin. She reported improvements in her symptoms gradually for a few days but eventually succumbed to the disease. Although generalized LAP is a rare initial presentation of SLE, it should be included in the differential diagnosis of the disease.

## Introduction

Systemic lupus erythematosus (SLE) is a major multisystemic autoimmune disease. It has many clinical manifestations, the severity of which ranges from mild (skin involvement) to fatal complications such as lupus cerebritis [central nervous system (CNS) involvement], lupus nephritis, and pericardial effusion. The incidence and prevalence of SLE vary among different countries. The global prevalence of SLE ranges from 20 to 150 cases per 100,000 persons [1]. SLE is more prevalent in females than males due to the hormone estrogen [2]. The etiology of SLE is multifactorial, including genetic, hormonal, and environmental factors [1]. In most patients, SLE ramifies itself chiefly through hematologic, renal, and cerebral manifestations. During the early course of the disease, the symptoms generally include fever, fatigue, weight loss, joint involvement, mucocutaneous symptoms, as well as pulmonary and ophthalmic involvement. The conventional treatment of SLE includes non-steroidal anti-inflammatory drugs (NSAIDs), antimalarials, glucocorticoids, and immunosuppressive agents [1]. In this report, we present the case of a 23-year-old female who was diagnosed with SLE with multi-organ involvement. The case was complicated by lupus cerebritis, lupus nephritis, and pericarditis with massive pericardial effusion. The case posed a diagnostic challenge as it initially presented with generalized lymphadenopathy (LAP), which is not frequently seen as an initial presentation of SLE.

## Case presentation

A 23-year-old female was brought to the emergency department with complaints of recurrent high-grade fever, seizures and, altered mental state (AMS). She had been seeking treatment from a local practitioner for a previously diagnosed tuberculous pericardial effusion, which had not yet resolved. Her past medical history included iron deficiency anemia, stomatitis, right mastitis, and she had been on anti-tubercular treatment (ATT) for the past 45 days. She had a family history of tuberculosis but denied having diabetes, hypertension, or asthma. She had suffered five to six episodes of abnormal body movements and altered sensorium before being admitted to the hospital. On admission, she had a fever of 101 ℉, tachycardia with 180 beats/minute, respiratory rate of 24 breaths/minute, blood pressure of 110/70 mmHg, and low oxygen saturation of 85% on ambient air. The patient's hematological and biochemical parameters are shown in Table 1 and Table 2 respectively. On physical examination, her lymph nodes were found to be tender and painful, and she had generalized LAP, involving the right-sided cervical, axillary, bilateral inguinal, and inter-trochanteric lymph nodes. The LAP had started as a swelling below the medial aspect of the right chin; the swelling had then progressed to the medial aspect of the right elbow and finally evolved to be generalized LAP. She also presented alopecia and malar rash. Her respiratory examination revealed the presence of bilateral crepitus. Her deep tendon reflexes were present but were depressed. Her CNS examination revealed a Glasgow Coma Scale (GCS) score of 8 (eye: 2, verbal: 2, motor: 4). Neck rigidity was also present. She was administered IV methylprednisolone. However, the LAP did not resolve. Later on, she was started on IV glucocorticoids and phenytoin, along with the continuation of ATT.

**Table 1:** Hematological parameters of the patient at the time of admission

Parameter	Reference range	Day 1	Day 5
Erythrocyte sedimentation rate, mm/hr	0.00-20.00	53	-
Hemoglobin, g/dL	12-16.5	6.6	6.5
Total leucocyte count, cells/mm^3^	4,000-10,000	18,200	12,700
Platelet count, cells/mm^3^	150,000-450,000	104,000	53,000
Red blood cell count, x 10^6 ^cells/mm^3^	3.8-4.8	2.46	2.55
Mean corpuscular volume, fL	80-100	64.2	65
Mean corpuscular hemoglobin, pg	27-32	26.9	25.5
Mean corpuscular hemoglobin concentration, g/dL	32-35	42	39.1
Packed cell volume, %	36-46	15.8	16.6

**Table 2:** Biochemical parameters of the patient at the time of admission

Parameter	Reference range	Day 1	Day 5
Anti-ds DNA antibody, IU/mL	<30.00	533.77 (positive)	-
C-reactive protein, mg/dL	<0.50	16.00	-
Serum protein, g/dL	6.0-8.3	5.6	6.5
Serum albumin, g/dL	3.8-5.5	2.6	3.3
Serum urea, mg/dL	13-43	42	-
Serum creatinine, mg/dL	0.6-1.2	1.1	1.6
Serum bilirubin (total), mg/dL	0-1.2	0.6	0.6
Serum bilirubin (direct), mg/dL	0-0.2	0.2	0.2
Serum bilirubin (indirect), mg/dL	0.2-0.7	0.4	0.4
Serum sodium, meq/L	137-150	141.8	152
Serum potassium, meq/L	3.5-5.3	4.33	4.1
Serum calcium, meq/L	4.5-5.5	4.88	5.20

**Figure 1:**
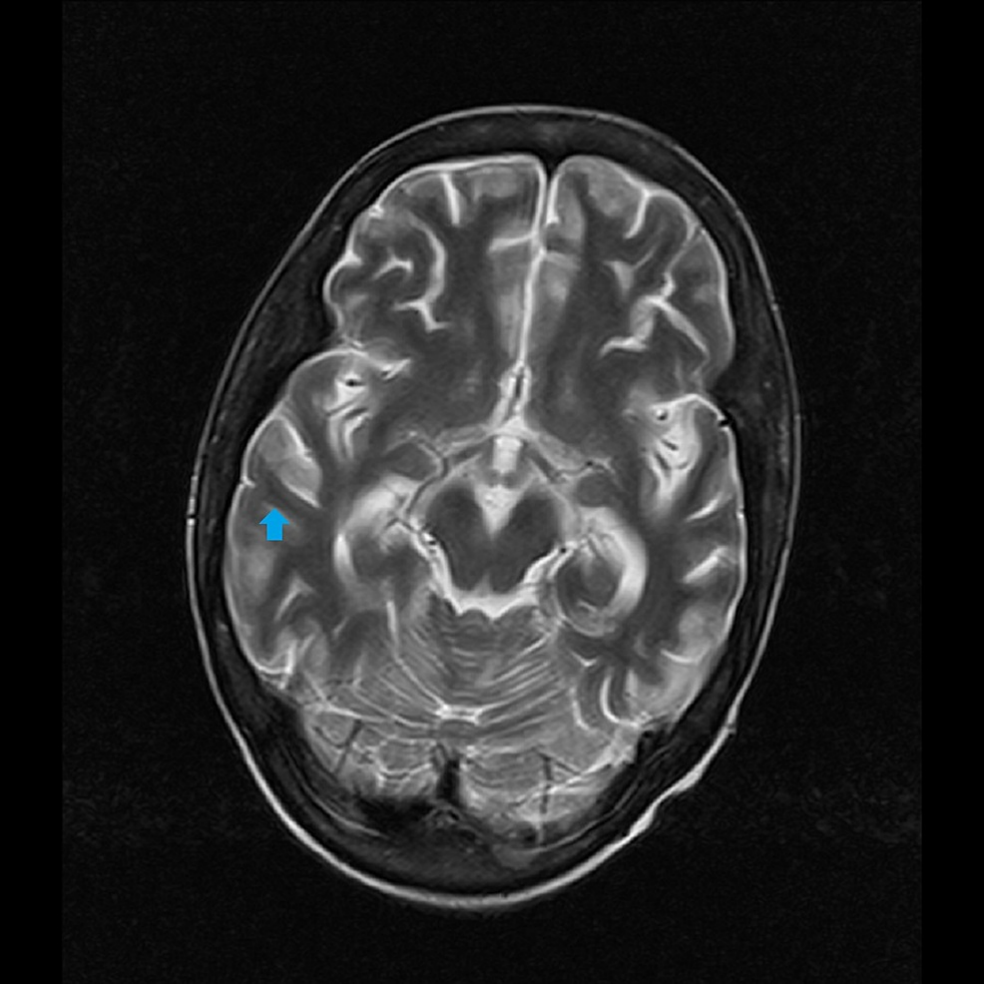
T2-weighted MRI of the transverse section of the brain showing enhanced brain parenchyma (arrow) MRI: magnetic resonance imaging

The patient's urine examination revealed proteinuria (total protein: 226.90 mg/dL; creatinine: 86.96 mg/dL; protein-creatinine ratio: 2.61), macroscopic hematuria with 15-20 red blood cells/high power field, and was indicative of lupus nephritis. Her cerebrospinal fluid (CSF) examination was suggestive of meningitis (total protein: 270.40 mg/dL; cell count: 10/mm^3^; all mononuclear and glucose: 56.21 mg/dL). Her MRI scan of the head revealed enhancements in the brain parenchyma, signifying inflammation and indicating lupus cerebritis (Figure 1).

**Figure 2:**
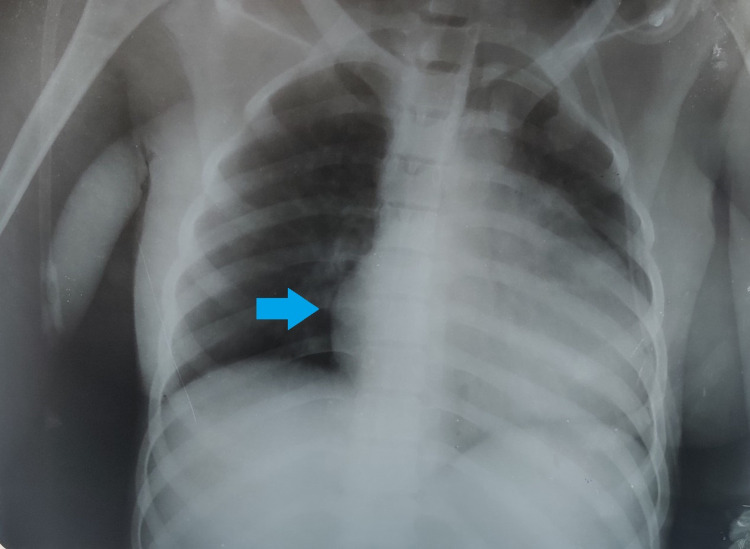
Plain chest X-ray of the patient showing bilaterally enlarged cardiac silhouette (arrow)

Her chest X-ray revealed a bilaterally enlarged cardiac silhouette (Figure 2), which raised suspicion for pericardial effusion. Her subsequent echocardiography confirmed our suspicion. Her anti-double stranded DNA (anti-dsDNA) was positive, and the anti-smooth muscle antibody (ASMA) was negative. Her immunofluorescence assay of pericardial fluid was positive for anti-nuclear antibodies (ANAs) with cytoplasmic dense fine-speckled pattern and intensity of 2+ (mildly positive) with a dilution of 1:80. In light of these findings, we concluded that her pericardial effusion was a complication of SLE rather than one caused by tuberculosis as diagnosed earlier by her local practitioner. According to the European League Against Rheumatism (EULAR) and the American College of Rheumatology (ACR) 2019 diagnostic criteria for SLE, a score of 10 or more must be considered as indicative of SLE. In our case, the score was 37, which is much more than what is needed to satisfy the criteria.

Even though the patient responded well to the treatment initially, she succumbed to the disease after one week of treatment.

## Discussion

SLE is a multisystemic autoimmune disorder that can affect any organ in the body; it is more prevalent among females than males, one of the possible causes being the hormone estrogen. The female-to-male ratio for adults is 7-15:1 [3,4]. Many infectious and non-infectious diseases can cause LAP. Generalized/peripheral LAP is not considered to be a specific symptom of SLE. According to the EULAR/ACR 2019 diagnostic criteria for SLE, LAP is not listed as a diagnostic criterion for SLE [5]. Though the exact prevalence of LAP in SLE is unknown, there have been a few case reports where LAP has been reported as the first presenting feature in SLE. Thus, generalized LAP as the presenting feature in SLE patients is relatively uncommon [6]. Hence, when LAP presents as the first clinical feature, the diagnosis becomes quite problematic, as in our case. Thrombocytopenia is reported in 20-40% of patients with SLE. The possible pathophysiology for thrombocytopenia is the clearance of platelets by anti-platelet antibodies [4]. Lupus nephritis is among the most severe complications of SLE; it may lead to kidney failure and is associated with a high risk of mortality. Proteinuria is reported in all patients with lupus nephritis and can be the only clinical finding besides normal or elevated serum creatinine levels. However, lupus nephritis can be classified into six types based on renal biopsy results [7]. The renal biopsy also helps determine the severity of lupus nephritis. In our case, the patient presented with marked proteinuria and elevated serum creatinine levels (Table 2). Cardiac complications affect 15-50% of all SLE patients, the most common one being pericarditis. A massive pericardial effusion as a presenting feature in SLE is not generally common. However, it is usually associated with kidney complications [8]. The psychiatric and neurological symptoms associated with SLE are termed lupus cerebritis and include depression, seizures, AMS, psychosis, and delirium. The clinical features that our patient presented with (seizures and AMS) and the subsequent MRI findings confirmed that she had cerebritis. Cases with such diverse presenting complications are extremely rare. In our case, three fatal complications - nephritis, cerebritis, and pericarditis - were involved, along with various other specific and non-specific clinical features that complicated the case. Managing cases like these on an individualized basis is complex and needs a multidisciplinary approach. The details of the standard treatment for SLE are presented in Table 3. Besides the standard treatment and phenytoin, the patient's ATT was also continued.

**Table 3:** Systemic therapy for SLE*** *In patients who do not respond adequately to HC and GC, with residual disease activity. **In patients with intolerance/contraindications to immunosuppressants. ***[9] SLE: systemic lupus erythematosus

	Mild/constitutional symptoms of SLE and no vital organ is affected	Severe symptoms of SLE and no vital organ is affected	Multi-organ damage and thrombocytopenia are present
Basic therapy	Hydroxychloroquine (HC); addition of methotrexate or azathioprine in case the patient does not respond to hydroxychloroquine		
Induction therapy	Low-dose oral glucocorticoids (GC)	Medium-dose oral glucocorticoids	High-dose IV glucocorticoids
		Immunosuppressants, monoclonal antibodies (e.g., belimumab*, rituximab**)	

## Conclusions

LAP can be associated with several etiologies. Although generalized LAP as the initial presentation of SLE is rare, it should be included in the differential diagnosis of the condition. In our case, the patient presented with generalized LAP, which made the diagnosis hard to narrow down to SLE. SLE can affect almost every organ in the body. In our case, SLE was complicated by nephritis, cerebritis, and pericarditis, along with the involvement of other tissues. We treated the patient with IV glucocorticoids (methylprednisolone) and phenytoin for seizures besides ATT for tuberculosis. Initially, the patient responded well to the treatment, but she succumbed to the disease after one week of treatment.
